# Automatically Detecting Failures in Natural Language Processing Tools for Online Community Text

**DOI:** 10.2196/jmir.4612

**Published:** 2015-08-31

**Authors:** Albert Park, Andrea L Hartzler, Jina Huh, David W McDonald, Wanda Pratt

**Affiliations:** ^1^ Department of Biomedical Informatics and Medical Education School of Medicine University of Washington Seattle, WA United States; ^2^ Group Health Research Institute Seattle, WA United States; ^3^ Department of Biomedical Informatics University of California San Diego San Diego, CA United States; ^4^ Human Centered Design & Engineering University of Washington Seattle, WA United States; ^5^ The Information School University of Washington Seattle, WA United States

**Keywords:** UMLS, natural language processing, automatic data processing, quantitative evaluation, information extraction

## Abstract

**Background:**

The prevalence and value of patient-generated health text are increasing, but processing such text remains problematic. Although existing biomedical natural language processing (NLP) tools are appealing, most were developed to process clinician- or researcher-generated text, such as clinical notes or journal articles. In addition to being constructed for different types of text, other challenges of using existing NLP include constantly changing technologies, source vocabularies, and characteristics of text. These continuously evolving challenges warrant the need for applying low-cost systematic assessment. However, the primarily accepted evaluation method in NLP, manual annotation, requires tremendous effort and time.

**Objective:**

The primary objective of this study is to explore an alternative approach—using low-cost, automated methods to detect failures (eg, incorrect boundaries, missed terms, mismapped concepts) when processing patient-generated text with existing biomedical NLP tools. We first characterize common failures that NLP tools can make in processing online community text. We then demonstrate the feasibility of our automated approach in detecting these common failures using one of the most popular biomedical NLP tools, MetaMap.

**Methods:**

Using 9657 posts from an online cancer community, we explored our automated failure detection approach in two steps: (1) to characterize the failure types, we first manually reviewed MetaMap’s commonly occurring failures, grouped the inaccurate mappings into failure types, and then identified causes of the failures through iterative rounds of manual review using open coding, and (2) to automatically detect these failure types, we then explored combinations of existing NLP techniques and dictionary-based matching for each failure cause. Finally, we manually evaluated the automatically detected failures.

**Results:**

From our manual review, we characterized three types of failure: (1) boundary failures, (2) missed term failures, and (3) word ambiguity failures. Within these three failure types, we discovered 12 causes of inaccurate mappings of concepts. We used automated methods to detect almost half of 383,572 MetaMap’s mappings as problematic. Word sense ambiguity failure was the most widely occurring, comprising 82.22% of failures. Boundary failure was the second most frequent, amounting to 15.90% of failures, while missed term failures were the least common, making up 1.88% of failures. The automated failure detection achieved precision, recall, accuracy, and F1 score of 83.00%, 92.57%, 88.17%, and 87.52%, respectively.

**Conclusions:**

We illustrate the challenges of processing patient-generated online health community text and characterize failures of NLP tools on this patient-generated health text, demonstrating the feasibility of our low-cost approach to automatically detect those failures. Our approach shows the potential for scalable and effective solutions to automatically assess the constantly evolving NLP tools and source vocabularies to process patient-generated text.

##  Introduction

The Internet pervades our everyday life, including health care [1]. For instance, patients increasingly use the Internet for health information and peer support. In 2005, 80% of Internet users searched for health information online [2]. Just 6 years later, a quarter of Internet users living with a chronic condition sought information online from a peer with a similar condition [3]. Similarly, applications that make use of data generated by patients are increasing [4]. For example, micro-blogging (eg, Twitter) has been used to improve natural disaster and emergency response situations [5], and patient-generated data on the PatientsLikeMe website has been used to evaluate the effectiveness of a drug [6]. Moreover, patients have voiced great potential benefits of such patient-generated data with respect to their treatment decisions, symptom management, clinical management, and outcomes [7,8]. However, in these instances, the use of patient-generated data required manual analysis of textual data. Although these methods provide value, manual analysis does not scale to the growing size of patient-generated health data online. Moreover, for many research activities, the overwhelming amount of data remains a challenge.

One scalable approach to process text-based patient-generated data is natural language processing (NLP). An increasing number of researchers studying patient-generated text, such as in online health communities, have used statistical methods based on manually annotated datasets [9-15]. Utilizing statistical methods, researchers extracted cancer patient trajectories from patients’ posts [9], estimated the level of social support in an online breast cancer community [10], predicted adverse drug reactions from health and wellness Yahoo! Groups [11], identified medically relevant terms [12], classified addiction phases [13], predicted individual at risks for depression [14], and discovered patient posts in need of expertise from moderators [15]. These methods can be highly effective in a given online community, but they either require tremendous upfront effort to manually annotate or do not provide semantic connections. Furthermore, maintenance and generalizability remain as major challenges for such statistical methods.

Existing biomedical NLP tools have the potential to be used immediately and promise to provide greater generalizability than statistical approaches while providing semantic connections. Researchers have developed various NLP techniques and applications in the biomedical domain. For example, the Clinical Text Analysis and Knowledge Extraction System (cTakes) [16] was developed to map concepts to medical ontologies from clinical notes. cTakes is specifically trained for clinical domains and consists of NLP components that can be executed in sequence. Also, the National Center for Biomedical Ontology [17,18] annotator identifies a term and maps it to ontological concepts from multiple knowledge resources to allow the use of integrated knowledge. Other applications have been developed primarily for specific uses, such as Medical Language Extraction and Encoding System (MedLEE) [19], whose goal pertains to identifying specified conditions in radiology reports. However, MedLEE was later adapted as a decision support system for Columbia-Presbyterian Medical Center [20] and as a phenotypic information extractor (BioMedLEE) [21] from biomedical literature.

One of the most widely regarded NLP applications in biomedicine is MetaMap [22], which was developed by the National Library of Medicine (NLM). MetaMap uses computational linguistic techniques to identify words or phrases in text and map them to concepts in the NLM’s Unified Medical Language System (UMLS). The UMLS is a collaborative effort to enable semantic interoperability among systems by connecting more than 1.3 million concepts from more than 100 biomedical vocabularies [23,24]. Three knowledge sources enable applications to utilize the UMLS: (1) the Metathesaurus, which connects synonymous concepts across vocabularies, (2) the Semantic Network, which is a hierarchical network of semantic types that are linked to every concept in the Metathesaurus, and (3) the SPECIALIST Lexicon, which provides the lexical information needed for NLP tools. Thus, MetaMap provides a semantic link between the words or phrases in text and a structured vocabulary that is used by many applications in biomedicine.

However, MetaMap and many other traditional biomedical NLP tools were developed to process biomedical literature and clinical notes, rather than patient-generated text in online communities. One of the biggest challenges in applying these biomedical NLP tools to a different type of text is the difference in vocabulary. For example, Zeng et al recognize differences in the vocabulary used by patients and clinicians [25]. Smith and Wicks manually evaluated patient-generated text from PatientsLikeMe and found that over 50% of patient-submitted symptoms did not map to the UMLS due to issues like misspellings and slang [26]. Although Keselman et al [27] reported fewer cases of unmapped terms from patient-generated online community posts than Smith and Wicks [26], the researchers recognize this remaining challenge as a significant problem.

Recognizing the differences in vocabulary, a number of efforts to expand the UMLS to include patient-generated text have been reported [25,27-31]. One of the biggest efforts is the open-access Collaborative Consumer Health Vocabulary Initiative (CHV) [25,27,31]. CHV is a collaborative effort to address differences in terminology by including layman-friendly terminology that is familiar to patients [25]. Although the terminology difference could theoretically be addressed by expanded vocabularies, it is questionable whether CHV can fully address other issues of patient-generated text, such as misspellings, community nomenclature, and Internet-oriented writing styles. To address this issue, Elhadad et al applied an unsupervised, semantics-based methods to detect community nomenclature including typical misspellings [32]. Although the method is domain-independent, it accounts for only three semantic types.

The effort to process patient-generated text, such as email [29,30] and search queries [27,28], using biomedical NLP tools has also been reported. For example, Brennan and Aronson processed patient-authored emails using MetaMap and showed the potential of processing this patient-generated, informal text to identify UMLS concepts [29]. However, Brennan and Aronson identified only three types of errors: (1) overly granular parsing of phrases into separate terms (eg, splitting of the phrase “feeling nauseous”), (2) inappropriate mappings that are simply nonsensical or incorrect for the context (eg, a verb “back” being mapped to “body location or region back”), and (3) mismatches resulting from terms and semantic types having more than one meaning (eg, confusion between “spatial concept right” and “qualitative concept right”) [29]. Zeng et al have mapped the UMLS concepts to patients’ Internet search queries [28], and the study highlighted the difference between terminology structures in UMLS and mental model of patients.

These prior studies show that many have worked to improve biomedical NLP tools to process patient-generated text. As NLP technologies and source vocabularies continue to evolve, we need easy, low-cost methods to systematically assess the performance of those tools. Traditionally in NLP, evaluations involve a great deal of manual effort, such as creating a manually annotated dataset. Moreover, a new evaluation for different types of text requires additional annotated datasets, thus maintenance can often be difficult. Recognizing the potential benefits of performing a low-cost assessment of NLP tools, we explore automated methods to detect failures without producing annotated datasets. Given MetaMap’s long history of use in biomedical contexts, its configurability, and its scalability, we apply our failure detection tool to MetaMap in processing patient-generated text from an online cancer community to demonstrate the feasibility of automatically detecting occurrence of failures. We first present the dataset and MetaMap configuration, followed by the specific methods and results for (1) characterizing failure types, (2) automated failure detection, and (3) manual performance evaluation of our automated failure detection approach.

## Methods

### Online Community Dataset and MetaMap Configuration

Our dataset consists of community posts from the CancerConnect website, an online cancer community for cancer patients, their families, friends, and caregivers to exchange support and advice. The dataset consists of a total of 2010 unique user members and 9657 user member–generated posts from March 2010 to January 2013.

We processed the online community posts with MetaMap version 2011AA and configured the word sense disambiguation feature and included only the top-ranked concept from the output. In the default setting, MetaMap suggests a number of candidate concepts with candidate scores indicating relationships among concepts found in the text. However, in real-world usage on large amounts of text, considering multiple suggestions for each processed term could be overwhelming to assess manually. Thus, we assessed only the top-ranked scored concept to simulate how MetaMap would be used in real-world settings. However, we used default settings for all other options for generalizability. A single mapped term/concept served as the unit of analysis.

### Methods for Characterizing Failures

To characterize the types of failures, we assessed MetaMap’s output collaboratively through iterative rounds of manual review among the five authors. We reviewed the output following an open coding process [33] to identify emerging themes grounded in data. Because we did not know all possible failure types, we chose to use an inductive coding process, rather than a structured, reductive content analysis approach. In each iteration, we processed different patient-generated posts, and each author independently and manually evaluated the same sets of mapped terms by examining the corresponding UMLS concept definitions and semantic types. Then as a group, we reached a consensus through discussion when different verdicts were made. Based on the list of inaccurate mappings, we grouped each inaccurate mapping into failure types and went on to identify potential causes within each failure type through the open coding process. This second step addressed the gaps in previous literature by identifying a number of causes of the failure types and providing information needed to detect these failures automatically.

### Results for Characterizing Failures

#### Overview

From our manual review, we characterized three types of failure: (1) boundary failures, (2) missed term failures, and (3) word ambiguity failures. A boundary failure occurred when a single coherent term was incorrectly parsed into multiple incomplete terms. A missed term failure occurred when a relevant term had not been identified. A word sense ambiguity failure occurred when a relevant term was mapped to a wrong concept. Within these three failure types, we discovered 12 causes of failures. In the sections below, we describe each type of failure and then identify potential causes within each failure type.

#### Boundary Failures

Boundary failures, in which a single coherent term is incorrectly parsed into multiple terms, are well documented in biomedical NLP literature [26,29,34-36]. In this literature, boundary failures are referred to as overly granular parsing [29] or split phrasing [34]. Our analysis expands our understanding with boundary failures associated with patient-generated text.

Our patient-generated text contained extensive descriptive phrases (eg, “feeling great”) and colloquial language (eg, “chemo brain”), contrasting with typical biomedical text that usually contained concepts from standard terminologies. Theoretically, boundary failures can result from standard medical terminologies. However, descriptive phrases and colloquial language highlight the parsing problem of biomedical NLP because colloquial language and descriptive phrases that patients use in online health communities cannot all be included in the UMLS. For instance, UMLS included “feeling sick” as a synonym of a concept, although a similar descriptive phrase “feeling great” was not included in the UMLS. Consequently in our analysis, “feeling sick” was recognized as one concept, while “feeling great” was parsed into two separate terms “Emotions” and “Large” delivering different interpretations than intended.

Boundary failures also occurred even when proper concepts were available in the UMLS. For instance, a colloquial term “chemo brain” was commonly used to describe the single concept of cognitive deterioration of cancer patients after chemotherapy. In our analysis, the term was recognized as two UMLS concepts—“chemotherapy” and “brain-body part”—even though UMLS contained a concept for “chemo brain”. From our experience, we inferred that the lack of colloquial language and descriptive phrases concepts in the UMLS as well as standard medical terminologies parser were causing boundary failure when processing patient-generated text.

#### Missed Term Failures

##### Overview

Missed term failures occurred when a relevant term was not identified [26,34]. We extended the literature by identifying two causes of missed term failures associated with patient-generated text: (1) community-specific nomenclature and (2) misspellings.

##### Community-Specific Nomenclature

Community-specific nomenclature refers to members of a community using terms that either are commonly used in a different way elsewhere or not commonly used at all. In online communities, members frequently create their own nomenclature that, over time, can become vernacular that is well understood in the community [37]. Community nomenclature poses unique challenges and opportunities for NLP.

In particular, community nomenclature regularly referred to relevant health-related content but resulted in three major challenges. First, many of the community-specific terms were not found in the UMLS. For instance, “PC” referred to “Prostate Cancer”; however, this acronym was not contained in UMLS. Second, community nomenclature was typically context and community-specific. For instance, the acronym “BC” was used for “before cancer”, “blood count”, or “breast cancer” depending on the context. This type of ambiguous usage was also seen with commonly accepted abbreviations. For instance, “rad” was a common abbreviation for “radiation therapy” in the cancer community, but “rad” could also be used for “radiation absorbed dose”, “reactive airway disease”, “reactive attachment disorder”, or “RRAD gene” depending on the community. Third, novel abbreviations and acronyms constantly showed up in our data, similar to what researchers of online communities found [37]. For instance, our dataset included newly emerged acronyms that were not included in the UMLS, such as “LLS” and “PALS” for “Leukemia and Lymphoma Society” and “Patient Advice and Liaison Service”, respectively.

##### Misspellings

Previous research showed that patients made more medically related misspellings at a significantly higher rate compared to clinicians [25]. Misspellings in our dataset included typographical errors (eg, “docotor”), phonetic errors that could be associated with lack of familiarity with medical terms (eg, “byopsi” and “methastasis”), and colloquial language errors (eg, “hooooooot flash”). Biomedical NLP techniques were typically developed using the correct spelling in training models, thus relevant but misspelled terms were often unrecognized. These unrecognized terms comprised a type of missed exact match [34] that consequently become false negatives—terms that should have been recognized but were missed. Although previous research in health information query investigated methods to address misspellings of patient-generated medical terms [38,39], those methods had limitations because they required correctly spelled medical terms in the database and manual selection of terms among recommended terms.

#### Word Sense Ambiguity Failures

The most prevalent failure was word sense ambiguity, which occurred when a term was mapped to the wrong concept because the two concepts are spelled the same way, share the same acronym (eg, “apt”, an acronym used for appointment was mapped to organic chemical “4-azido-7-phenylpyrazolo-(1,5a)-1,3,5-triazine”), or were spelled the same as one of their acronyms (eg, a verb “aids” was mapped to “Acquired Immunodeficiency Syndrome”). This failure had been identified in previous research [26,29,34-36], but these studies did not examine the causes of this failure. From our analysis, we identified nine causes of failure associated with processing patient-generated text: (1) abbreviations and contractions, (2) colloquial language, (3) numbers, (4) email addresses and Uniform Resource Locators (URL)s, (5) Internet slang and short message service (SMS) language, (6) names, (7) the narrative style pronoun “I”, (8) mismapped verbs, and (9) inconsistent mappings (by word sense disambiguation feature). In the following sections, we describe each cause of word sense ambiguity failures in detail and identify associated semantic types where applicable.

##### Abbreviations and Contractions

Frequent use of standard abbreviations and contractions was common in our online health communities. Online community members frequently used contractions such as “I’d” or abbreviations such as “i.e.” in their text. Although the use of these shortened forms was common in informal text, it could be a source of errors for many NLP tools. For example, MetaMap maps “I’d” to “Incision and drainage” and mapped “i.e.” to “Internal-External Locus of Control Scale” due to partial matches with synonyms. Also, MetaMap was inconsistent with some of its correct mappings for abbreviations. For instance, abbreviations for some US states were mapped correctly (eg, “AK” and “WA”), whereas others were often missed even though they were in the UMLS (eg, “CA” and “FL”) or were mismapped (eg, Virginia was mapped to “Alveolar gas volume” when written as “V.A.”).

##### Colloquial Language

Colloquial language, such as “hi” was prevalent in our dataset and caused many failures. Although these terms are obvious to human readers, we found they were often mapped to incorrect terms in the UMLS. For instance, our previous example “hi”, rather than being left unmapped, was mapped to “Hawaii”, “ABCC8 gene”, or “AKAP4 gene” because “hi” was a synonym for all three concepts. In our analysis, this failure was found with many semantic types; however, terms mapped to the semantic type of “Gene or Genome” were particularly troublesome because of their unusual naming conventions.

##### Numbers: Dates, Times, and Other Numbers Not Indicating Disease Status

Our online community posts often contained numbers that convey important information, such as a patient’s disease status (eg, “stage 3 breast cancer”). Other times, numbers conveyed more logistical information, such as time of day and dates, which were misinterpreted. For instance, in the phrase, “I got there at 4:12pm”, “12pm” was mapped to “Maxillary left first premolar mesial prosthesis” because it was a complete match for one of its synonyms in the UMLS. Numbers that were used to convey diagnostic information were crucial for the identity of many community members, and such information was often included in an automated signature line (eg, “stage 2 grade 3 triple negative breast cancer”) at the end of posts. Numbers indicating dates and times often resulted in false positives, whereas health status numbers often resulted in a different failure type (ie, boundary failure caused by splitting a phrase). We saw this type of failure across many different semantic types, including “Amino Acid, Peptide, or Protein”, “Finding”, “Gene or Genome”, “Intellectual Product”, “Medical Device”, “Quantitative Concept”, and “Research Activity”.

##### Email Addresses and URLs

Online community members frequently mentioned URLs and email addresses in our dataset. They often pointed to websites that they found useful and gave out email addresses to start private conversations. Parts of email addresses and URLs were incorrectly mapped in our analysis. For instance, “net” at the end of an email address was often mapped to the “SPINK5 gene” because one of its synonyms was “nets”. Also, “en”, a language code that referred the English language in URLs, incorrectly mapped to “NT5E gene” because one of its synonyms was “eN”.

##### Internet Slang and SMS Language

Internet slang and SMS language, such as “LOL” (ie, “laugh out loud” or “lots of love”) or “XOXO” (ie, hugs and kisses) are highly prevalent in online community text but not in typical biomedical texts. Although these terms should be obvious to human readers, our analysis showed that Internet slang and SMS language were often mapped to incorrect biomedical terms in the UMLS. In particular, Internet slang and SMS language were often mistaken for gene names, such as the mapping of “LOL” to the LOX1 gene and “XO” to the XDH gene. To manage the different variations of concepts, the UMLS included many synonyms of terms. Varieties of these synonyms overlapped with commonly used Internet slang and SMS language resulting in word sense ambiguity failure.

##### Names: First, Last, and Community Handles

The use of names is also prevalent in online community posts, particularly when posts address specific individuals. Community members also often include their first names in a signature line and call out other members by first names or community handles. In our analysis, common first names were often mistaken for UMLS concepts, such as “Meg” being mistaken for “megestrol”, “Rebecca” for “becatecarin”, “Don” for “Diazooxonorleucine”, and “Candy” for “candy dosage form”. Each individual name was a complete match for one of the UMLS concepts. We identified these mismatches across multiple semantic types, including “Antibiotic”, “Biomedical or Dental Material”, “Clinical Attribute”, “Diagnostic Procedure”, “Disease or Syndrome”, “Finding”, “Hormone”, “Injury or Poisoning”, “Laboratory Procedure”, “Mental Process”, “Pathologic Function”, “Pharmacologic Substance”, and “Sign of Symptom”.

##### Narrative Style of Pronoun “I”

Patients share a wide variety of personal experiences in narrative form in online health communities. Thus, the use of the pronoun “I” is prevalent in community posts but is a source of misinterpretation. For example, over the course of the study we discovered that “I” is typically mapped to either “Blood group antibody I” or “Iodides”, which belong to “Amino Acid, Peptide, or Protein”, “Immunologic Factor”, or “Inorganic Chemical” semantic types.

##### Mismapped Verbs

One of the most fundamental components of NLP tools is a part-of-speech (POS) tagger, which marks up words with their corresponding POS (eg, verb, noun, preposition) in a phrase, sentence, or paragraph. POS taggers are commonly used in NLP and have many different applications, such as phrase parsers. In our analysis, we discovered that MetaMap uses a POS tagger called MedPost SKR (Semantic Knowledge Representation) [40] to split text into phrases. However, it did not use the resulting POS information when mapping to the UMLS. Such POS failures could have been overlooked in previous studies using biomedical text due to the fact that words like “said” or “saw” were less prevalent in biomedical literature or even in clinical notes. For our online community dataset, MetaMap improperly mapped terms without discriminating between verbs and nouns. For instance, simple verbs used in past tense, like “said” and “saw”, were mapped as the acronym, “SAID” (ie, Simian Acquired Immunodeficiency Syndrome) and “saw” (ie, a medical device). Verbs in the present tense were also problematic. For instance, “bow” and “snap” were mapped to “Genu varum” and “Snap brand of resin”, respectively. We observed this type of failure across different semantic types, including semantic types where verbs were unexpected, such as “Antibiotic”, “Biomedical or Dental Material”, and “Pharmacologic Substance”.

##### Inconsistent Mappings

Two great strengths of the UMLS are its broad coverage of concepts and its capacity to distinguish among concepts in fine detail. This ability to provide the precise meaning of concepts is valuable for many applications. However, this feature also became a source for inconsistent mappings despite similar usage of terms in our analysis. For instance, the term “stage” was mapped to multiple concepts in our dataset. Community members often used the term “stage” to describe their cancer status (eg, *“*stage 4 ER+ breast cancer*”*). Despite the seemingly similar sentence structures and usage of the term in the sentence, our findings showed that MetaMap inconsistently mapped “stage” to different UMLS concepts. Six different semantic types were identified for the UMLS concepts mapping to “stage” (Table 1). This is a known failure of MetaMap [34]; however, the severity of the failures shows that addressing word sense disambiguation in patient-generated text may require particular attention.

**Table 1:** Word sense ambiguity failures: inconsistent mappings of stage by MetaMap.

Sample sentence	Mapped term	UMLS concept	Concept unique identifiers	UMLS semantic type
“My father was diagnosed with stage 2b pancreatic cancer”	stage 2b	Stage 2B	C0441769	Classification
“I'm stage 4 SLL and stage 2 CLL”	stage	Tumor stage	C1300072	Clinical attribute
“I was dx last year at age 46 with Stage 1”	Stage 1	Stage level 1	C0441766	Intellectual product
“Almost seven years ago I was diagnosed with stage 1 breast cancer at age 36 ½”	Stage breast cancer	malignant neoplasm of breast staging	C2216702	Neoplastic process
“My friend was just diagnosed with Stage IV cancer”	stage	Stage	C1306673	Qualitative concept
“My mom was diagnosed 11/07 with stage IV inoperable EC”	stage	Phase	C0205390	Temporal concept

### Methods for Automated Failure Detection

#### Overview

To explore automated methods for detecting the three types of failures we identified, we created a tool that applies combinations of dictionary-based matching [41-43] and NLP techniques [44-47]. We describe this detailed automatic detection process in the following sections.

#### Detecting Boundary Failures

Our tool detected failures caused by incorrectly splitting a phrase through a comparison of MetaMap’s MedPost SKR parser [40], a biomedical text parser, and the Stanford Parser [45] (a general-purpose parser). First, we collected all adjacent terms that MetaMap mapped but MedPost SKR had parsed separately. Second, we used the Stanford Parser to determine whether adjacently mapped terms were part of the same phrase. If adjacently mapped terms were part of the same phrase, the combined term could deliver a more precise meaning, while individually they often deliver different meanings [29,34]. We found this especially problematic if the combined term was a valid UMLS concept. For instance, we would collect “chemo brain” as a boundary failure caused by splitting a phrase. “Chemo” and “brain” were terms that appeared adjacent to one another in a sentence, and their combination—“chemo brain”—was a valid UMLS concept, but MetaMap split it into two separate terms. However, we also collected combined terms that were not in the UMLS because they were also cases of improperly splitting terms. Furthermore, the missing combined terms could provide valuable insight to completeness of the UMLS. For instance, both “double mastectomy” and “chemo curls” are important concepts that are frequently used by patients; however, these concepts are missing from the UMLS as shown in Table 2. The aforementioned steps to compare MetaMap’s MedPost SKR parser with the Stanford Parser can detect these important but missing terms. In our detection, we used the shortest possible phrase identified by the Stanford Parser. The Stanford Parser parsed phrases as structure trees to indicate grammatical relations. In the structure tree, a shorter phrase was often part of a longer phrase and delivered more coherent meanings compared to a longer phrase.

**Table 2:** Examples of splitting a phrase failure.

Sample sentence	Ideally mapped UMLS concept	First mapped term (UMLS concept name)	Second mapped term (UMLS concept name)
“My mom had unknown primary and it was a PET scan that helped them find the primary.”	PET/CT scan	PET (Pet Animal)	Scan (Radionuclide Imaging)
“It was removed and I have had stereotactic treatment along with 6 rounds of Taxol/Carbo completed in January 2012.” [sic]	Stereotactic Radiation Treatment	Stereotactic (Stereotactic)	Treatment (Therapeutic Aspects)
“Had 25 internal rad treatments (along with cisplatin on day 1 and 25).” [sic]	Therapeutic Radiology Procedure	Rad (Radiation Absorbed Dose)	Treatments (Therapeutic Procedure)
“I am Triple Negative BC and there are no follow-up treatments for us TN's.”	Triple Negative Breast Neoplasms	Triple (Triplicate)	Negative (Negative)
“My doc thinks I will probably end up having a double mastectomy”	None available	Double (Double Value Type)	Mastectomy (Mastectomy)
“I thought after 9 months my hair would be back but I have grown some type of hair that I am told is ‘chemo curls’.”	None available	Chemo (Chemotherapy Regimen)	Curls (Early Endosome)

#### Detecting Missed Term Failures

##### Overview

We identified two causes of missed term failures associated with processing patient-generated text. The following sections describe automatic detection of missed terms, specifically due to community-specific nomenclature and misspellings.

##### Community-Specific Nomenclature

Our tool detected missed terms due to abbreviations and acronyms in four steps. First, it ran MetaMap on the original text and then counted the total number of mappings. Second, it extracted common abbreviations and acronyms and their definitions using a simple rule-based algorithm [46], but where we manually verified the extracted terms. Third, it ran MetaMap again after replacing the extracted abbreviations and acronyms with their corresponding fully expanded terms. Finally, it calculated the difference in the total mappings between the original text and the updated text.

The simple rule-based algorithm by Schwartz and Hearst [46] has performed well in finding abbreviations and acronyms in documents [48,49]. We modified the algorithm to reflect typical writing styles of online community posts. The algorithm by Schwartz and Hearst uses (1) order of characters, (2) distance between abbreviations/acronyms and their definitions, and (3) presence of parentheses to find candidates for abbreviations/acronyms and their definitions. Our tool first identified completely capitalized words (with an exception of the last character due to pluralization) as candidate abbreviations/acronyms and then applied the algorithm to find its fully expanded form. Because online community members adopted community’s abbreviations/acronyms, we saved this information and applied to other posts written in the same community even when the definition was not available. For instance, in the sentence, “My mother was diagnosed with Stage 3 Esophageal cancer (EC) earlier this year - EC also counts smoking and alcohol as two major aggravating factors and is an aggressive cancer”, the poster defined EC once and then continued to use the acronym in place of esophageal cancer. MetaMap could map esophageal cancer but not EC. Our tool used this algorithm to detect EC and its fully expanded form, esophageal cancer, then replaced EC with “esophageal cancer” to ensure the concept could be identified by MetaMap.

##### Misspellings

Our tool detected the prevalence of missed terms due to misspelling using three steps. First, it ran MetaMap on the original text and counted the total number of mappings. Second, it ran MetaMap again after correcting possible misspellings using Google’s query suggestion service [50]. Finally, it calculated the difference in the mappings between the original text and the corrected text.

#### Detecting Word Sense Ambiguity Failure

We identified nine causes of word sense ambiguity failure associated with processing patient-generated text. In the following sections, we describe how to automatically detect the word sense ambiguity failures.

##### Abbreviations and Contractions

To detect word sense ambiguity failures due to abbreviations and contractions, we used an NLP tool called the Stanford POS Tagger [44], which assigns POS to terms in text. Our tool processed the data using the Stanford POS Tagger to count cases where a single mapped term was tagged with multiple POS. For instance, the Stanford POS Tagger would accurately tag “I’d” with two different POS, that is, the personal pronoun and modal.

##### Colloquial Language

Detecting word sense ambiguity failure caused by colloquial language is particularly challenging. We identified many of these failures by narrowing our focus to consider only the “gene or genome” semantic type because colloquial language failures were frequently mapped to this semantic type. Our tool automatically detected improperly mapped colloquial language by using an existing cancer gene dictionary—a list of genes known to be associated with cancer [43]—and counting the number of terms categorized as a “gene or genome” semantic type that were not in the cancer gene dictionary.

##### Numbers: Dates, Times, and Other Numbers Not Indicating Disease Status

To automatically detect improperly mapped dates and times, we implemented a number of rule-based regular expressions to detect times and dates that were not mapped as “Quantitative Concept” semantic type concepts. “Quantitative Concept” is the most appropriate semantic type based on how patients typically used numbers in our dataset. This resulted in counting the numbers mapped to “Amino Acid, Peptide, or Protein”, “Finding”, “Gene or Genome”, “Intellectual Product”, “Medical Device”, and “Research Activity”.

In our approach, we recognized two types of date or time expression that are problematic for MetaMap. The first type was a time expression containing the term “pm”. The second type was a string of numbers that has been typically used to describe age, date, or time duration. For instance, “3/4” indicating March fourth was mapped to a concept describing distance vision: concept unique identifier (CUI) C0442757. We used specific regular expressions that focused on numbers with “am” or “pm”, as well as a string of numbers with or without non-alphanumeric characters in between numbers to identify dates, times, and other numbers that do not indicate disease status.

##### Email Addresses and URLs

Our detection process for email addresses and URLs was completed using regular expressions to identify all the email addresses and URLs, and then we counted the number of terms that were mapped from email addresses or URLs. In our approach, we used specific regular expressions matching “@” and a typical structure of domain name (ie, a dot character followed by 2-6 alphabetic or dot characters) for identifying email addresses and “http” or a typical structure of domain name for identifying URLs.

##### Internet Slang and SMS Language

We detected improperly mapped Internet slang and SMS language using a 3-step process. First, we identified an Internet dictionary with a list of chat acronyms and text shorthand [41]. Second, we manually reviewed the list to remove terms that were also medical acronyms. In this process, we identified only three medical acronyms, “AML”, “CMF”, and “RX” and removed them from the list. Third, our tool automatically identified the terms in the text by matching them with the Internet slang/SMS language list.

##### Names: First, Last, and Community Handles

To identify improperly mapped names, we first combined a number of name dictionaries that consist of first names [42] with a list of community handles from our online community, CancerConnect. Then, our tool counted the number of mapped terms that matched one of the names in the combined list.

##### Narrative Style of Pronoun “I”

We identified a number of cases where the pronoun “I” was improperly assumed to be an abbreviation, such as for Iodine, because the NLP tool did not consider the contextual knowledge from the term’s POS. One of the most fundamental components of NLP tools is a POS tagger, which marks up words with their corresponding POS (eg, verb, noun, preposition) in a phrase, sentence, or paragraph. “I” as an abbreviation for Iodine should be recognized as a noun, whereas “I” meaning the individual should be recognized as a pronoun by a POS tagger. Our tool used data derived from the Stanford POS Tagger [44] to count cases where the pronoun “I” was mapped to either the “Blood group antibody I” or “Iodides” concepts. We noticed that the pronoun “I” was sometimes tagged as a foreign word. We included those cases in our counts because it was a failure of the Stanford POS Tagger.

##### Mismapped Verbs

To identify the improperly mapped terms without discriminating between verbs and nouns, we used POS information from the Stanford POS Tagger [44] to count cases where a mapped verb term belonged to a semantic type that did not contain verbs. The 34 semantic types (eg, “Activity” and “Behavior”) listed under the “Event” tree of the UMLS ontology could contain verbs; thus, we excluded verbs from these semantic types from our analysis. We considered all verbs in the “Entity” tree of the UMLS ontology as incorrect mappings. The “Entity” portion includes semantic types, such as “Biomedical or Dental Material”, “Disease or Syndrome”, “Gene or Genome”, “Medical Device”, “Pharmacologic Substance”, for which we do not expect verbs. Thus, our tool detected cases where verbs were associated with the “Entity” tree of the UMLS ontology.

##### Inconsistent Mappings

Detecting word sense ambiguity failures leading up to this section consisted of cases where terms were consistently mapped improperly. However, for other word sense ambiguity failures, MetaMap inconsistently mapped terms, both correctly and incorrectly. The inconsistency was the result of poor performance by MetaMap’s word sense disambiguation feature that was designed to select the best matching concepts out of many candidate concepts available in the UMLS. We detected inconsistent mappings by (1) assuming that patients used terms consistently, and (2) MetaMap accurately selecting the best matching concepts the majority of the time. For instance, in our online cancer community dataset, we assumed that patients always used the term “blood test” to convey the “Hematologic Tests” concept (CUI: C0018941), which was how MetaMap interpreted this term two thirds of the time, rather than the less frequent mapping to the “Blood test device” concept (CUI: C0994779). Based on these assumptions, we detected inconsistent mappings in two steps. First, we created a term frequency table based on a term’s spelling and its CUI. Second, assuming the most frequently mapped CUI was the correct concept, we counted the number of cases where the term was mapped to less frequent CUIs.

### Results for Automated Failure Detection

The automated methods detected that at least 49.12% (188,411/383,572) of MetaMap’s mappings for our dataset were problematic. Word sense ambiguity failures were the most widely occurring, comprising 82.22% among the total detected failures. Boundary failures were the second most frequent, amounting to 15.90% among the total detected failures, while missed term failures were the least common, making up 1.88% of the detected failures. Table 3 summarizes the identified failures as well as their causes and prevalence for automatic detection of MetaMap’s failure on processing patient-generated text. Our process showed the feasibility of automated failure detection; hence showing the types of failures that our tool could identify in similar datasets processed with biomedical NLP tools.

We found that word sense ambiguity failures were not mutually exclusive, and several cases had multiple causes. Thus, in Table 3, the sum of percentages for individual failures exceeded 100%. For instance, an acronym “OMG” used for “Oh My God” was incorrectly mapped to “OMG gene”. This particular failure was detected as both colloquial language as well as Internet slang and SMS language failures. To avoid redundant counts, we detected 154,904 unique counts of word sense ambiguity failure, making up 82.22% of failures. In Table 3, we show both individual counts/percentages as well as the total unique counts/percentages to provide a precise overview of word sense ambiguity failures. Although these failures were recognized in prior studies on MetaMap [26,29,34-36], the studies had not presented automated methods for detecting these failures.

We manually evaluated the performance of our failure detection tool in two parts: overall performance evaluation and individual component level performance evaluation.

**Table 3:** Detecting MetaMap’s failures on processing patient-generated text.

Failure type	Causes of failure	Count	Percentage of failure, %
1. Boundary failures	1.1 Splitting a phrase	29,965	15.90
2. Missed term failures	2.1 Community specific nomenclatures	1167	0.62
	2.2 Misspellings	2375	1.26
3. Word sense ambiguity failures	3.1 Abbreviations and contractions	416	0.22
	3.2 Colloquial language	4162	2.21
	3.3 Numbers	143	0.08
	3.4 Email addresses and URLs	1448	0.77
	3.5 Internet slang and SMS language	3442	1.83
	3.6 Names	10,061	5.34
	3.7 Narrative style of pronoun ‘I’	61,119	32.44
	3.8 Mismapped verbs	51,193	27.17
	3.9 Inconsistent mappings	29,308	15.56
	Total number of unique word sense ambiguity failures	154,904	82.22
Total number of unique failures		188,411	

### Methods for Performance Evaluation of Automated Failure Detection

We randomly selected 50 cases (ie, mappings) that our tool identified as incorrect mappings from each of the 12 causes of failures, totaling 600 cases that served as positive cases. We then randomly selected another 600 cases from the rest of the mappings not detected as incorrect mappings according to our tool to serve as the negative cases. We then mixed up the selected 1200 cases and manually assessed the accuracy of mappings through a blind procedure.

We also measured individual performance on each of the 12 detection techniques. We used the previously selected 600 negative cases and individual technique’s 50 positive cases to assess the performance. For boundary failure, we examined whether the mapped terms could deliver precise conceptual meaning independent of additional phrases. For missed term failure, we investigated whether the tool had accurately corrected the spellings and verified the results of the new mappings. For word sense ambiguity failures, we examined whether MetaMap appropriately mapped terms based on the rest of the context. The unit of analysis was a single mapping, and we evaluated our results using precision, recall, accuracy, and F1 score. Precision measures the proportion of predicted positive instances that are correct. Recall measures the proportion of positive instances that were predicted. Accuracy measures the percentages of correctly predicted instances among the total number of instances examined. F1 score is the weighted harmonic mean—reflecting both performance and balance—of precision and recall. In all measures, higher scores reflect better performance.

### Results for Performance Evaluation

#### Overview

Table 4 shows the performance of the automatic failure detection tool. The failure detection tool achieved overall precision, recall, accuracy, and F1 score of 83.00%, 92.57%, 88.17%, and 87.52%, respectively. At the individual component level, methods using dictionary-based matching or regular expression matching performed more accurately than methods using existing NLP techniques. In the following sections, we discuss findings of individual component of the automatic failure detection tool and its performance.

**Table 4:** Performance (in %) of automatic failure detection and its individual component.

Failure type	Causes of failure	Precision	Recall	Accuracy	F1 score
1. Boundary failures	1.1 Splitting a phrase	82.00	78.85	96.78	80.39
2. Missed term failures	2.1 Community specific nomenclatures	88.00	100.00	99.02	93.62
	2.2 Misspellings	80.00	93.02	97.88	86.02
3. Word sense ambiguity failures	3.1 Abbreviations and contractions	82.00	95.35	98.20	88.17
	3.2 Colloquial language	100.00	100.00	100.00	100.00
	3.3 Numbers	100.00	100.00	100.00	100.00
	3.4 Email addresses and URLs	100.00	100.00	100.00	100.00
	3.5 Internet slang and SMS language	100.00	100.00	100.00	100.00
	3.6 Names	66.00	100.00	97.21	79.52
	3.7 Narrative style of pronoun “I”	100.00	100.00	100.00	100.00
	3.8 Mismapped verbs	32.00	100.00	94.43	48.48
	3.9 Inconsistent mappings	66.00	53.23	92.80	58.93
Total		83.00	92.57	88.17	87.52

#### Boundary Failure

Our automatic failure detection tool identified 15.90% of the total failures as due to splitting a phrase. The performance evaluation of this task achieved precision, recall, accuracy, and F1 score of 82.00%, 78.85%, 96.78%, and 80.39%, respectively. It is important to note that a single concept can produce multiple split phrase failures. For instance, the phrase “stage 4 Melanoma” was mapped to three concepts: “stage”, “4”, and “Melanoma”. Two boundary failures occurred in this phrase. The first failure occurred between “stage” and “4”; the second failure occurred between “4” and “Melanoma”. By focusing on a pair of mapped terms at a time, we correctly identified two failures that occurred in the phrase “stage 4 Melanoma”. We considered only adjacent paired mappings because splitting a single coherent phrase into two or more UMLS concepts was clearly a more significant problem. However, split phrase failures could occur in non-paired mappings as well, and we are underestimating the prevalence of split phrases.

#### Detecting Community-Specific Nomenclature

Less than 1% of failures were due to community-specific nomenclature, and the automatic detection system achieved precision, recall, accuracy, and F1 score of 88.00%, 100.00%, 99.02%, and 93.62%, respectively. It should be noted that we underestimated the number of missed terms because the algorithm [46] can identify abbreviations or acronyms only if they were previously defined by members at some point. In addition, we would not recognize cases where MetaMap still missed the fully expanded term.

#### Detecting Misspellings

We automatically assessed that misspellings were responsible for 1.26% of failures. However, we observed few cases of incorrect assessment due to failures of Google’s query suggestion service. For instance, some medications were incorrectly recommended. “Donesaub”, a misspelling of “Denosumab” was mapped to “dinosaur”. Furthermore, even with correct recommendation, MetaMap did not always map to the right concept. For instance, “Wsihng” was correctly recommended to be “Wishing”, but MetaMap mapped it to “NCKIPSD gene”. Despite a few cases of incorrect assessment, the misspelling component performed relatively well, achieving precision, recall, accuracy, and F1 score of 80.00%, 93.02%, 97.88%, and 86.02%, respectively.

#### Detecting Abbreviations and Contractions

Improperly mapped abbreviations comprised less than 1% of failures. Although this was seldom, the automatic detection system performed relatively well, achieving precision, recall, accuracy, and F1 score of 82.00%, 95.35%, 98.20%, and 88.17%, respectively.

#### Detecting Colloquial Language

Incorrectly mapped “gene or genome” semantic types comprised 2.21% of failures, and the automatic detection system achieved precision, recall, accuracy, and F1 score of 100.00%, 100.00%, 100.00%, and 100.00%, respectively. With this process, we also detected terms like “lord” and “wish” that may not be perceived as colloquial language. Nevertheless, they were improperly mapped as “gene or genome” semantic type. It is also important to note that different disease-specific communities should utilize different gene dictionaries.

#### Detecting Numbers: Dates, Times, and Other Numbers Not Indicating Disease Status

Our automatic failure detection tool identified less than 1% of failures as improperly mapped numbers. The performance evaluation of this task achieved precision, recall, accuracy, and F1 score of 100.00%, 100.00%, 100.00%, and 100.00%, respectively. However, we are underestimating this failure prevalence because MetaMap improperly mapped more than half of the “Quantitative Concept” semantic type concepts in our dataset. We did not include this semantic type and underestimated this particular failure because few cases were correctly mapped.

#### Detecting Email Addresses and URLs

Improperly mapped email addresses or URLs comprised less than 1% of failures, and the automatic detection system achieved precision, recall, accuracy, and F1 score of 100.00%, 100.00%, 100.00%, and 100.00%, respectively. It is important to note that the basis for our manual assessments was how patients had intended to use the term. For instance, MetaMap mapped “org” at the end of a URL to “Professional Organization or Group” concept. Although assessment of such cases can be subjective, we followed the basic rule of reflecting patients’ intentions.

#### Detecting Internet Slang and SMS Language

A total of 1.83% of failures resulted from Internet slang and SMS language terms. Like other dictionary-based matching techniques, our automatic detection system performed relatively well, accomplishing precision, recall, accuracy, and F1 score of 100.00%, 100.00%, 100.00%, and 100.00%, respectively.

#### Detecting Names: First, Last, and Community Handles

We automatically assessed that names accounted for 5.34% of failures. However, the name dictionary matching did not perform as well as other dictionary-based matching components. We discovered that unique but popular names, such as “Sunday”, “Faith”, and “Hope” were incorrectly mapped when used as nouns in a sentence. The name dictionary component achieved precision, recall, accuracy, and F1 score of 66.00%, 100.00%, 97.21%, and 79.52%, respectively.

#### Detecting Narrative Style of Pronoun “I”

We found that 32.44% of failures resulted from pronoun “I”. Although the use of the pronoun “I” could be considered a part of colloquial language*,* we noted it as a different cause of failure due to its high frequency. The automatic detection system accomplished precision, recall, accuracy, and F1 score of 100.00%, 100.00%, 100.00%, and 100.00%, respectively.

#### Detecting Mismapped Verbs

We automatically assessed that mismapped verbs accounted for 27.17% of failures; however, the detecting mismapped verbs component performed poorly, achieving precision, recall, accuracy, and F1 score of 32.00%, 100.00%, 94.43%, and 48.48%, respectively. We discovered that although Stanford POS Tagger has identified verbs correctly, we made the false assumption that verbs did not belong to the entity part of the UMLS ontology. However, verbs like “lost” and “wait” belong to the “Functional Concept” semantic type, which is under the entity part of the UMLS tree. Thus, the detecting mismapped verbs component of our automatic failure detection tool incorrectly identified such verbs as failures.

#### Detecting Inconsistent Mappings

Our automatic failure detection tool identified 15.56% of the total failures due to inconsistent mappings. The performance evaluation of this task achieved precision, recall, accuracy, and F1 score of 66.00%, 53.23%, 92.80%, and 58.93%, respectively. We found two reasons for the relatively low precision. First, we did not account for cases where the most commonly mapped concept is not the correct mapping. For instance, in our dataset “radiation” was mapped to “radiotherapy research” (CUI: C1524021) two-thirds of the time when community members actually meant “therapeutic radiology procedure” (CUI: C1522449). We incorrectly assessed if less frequent mappings were accurate. Second, we missed cases when correct mappings do not exist. For instance, the verb “go” was incorrectly but consistently mapped as “GORAB gene”. In our automated failure detection analysis, our tool overlooked terms like “go” that were consistently mismapped.

## Results

We characterized (1) boundary failures, (2) missed term failures, and (3) word ambiguity failures and discovered 12 causes for these failures in our manual review. We then used automated methods and detected that almost half of 383,572 MetaMap’s mappings were failures. 82.22% of failures were word sense ambiguity. 15.90% of failures were boundary failure. 1.88% of failures were missed term failures. The automated failure detection achieved precision, recall, accuracy, and F1 score of 83.00%, 92.57%, 88.17%, and 87.52%, respectively.

##  Discussion

### Principal Considerations

We first discuss challenges of using out-of-the-box biomedical NLP tools, such as MetaMap, to process patient-generated text. We then discuss the contributions and wider implications of our study for research activities that need to manage the constantly changing and overwhelming amount of patient-generated data. We end with summarizing our contributions to the medical Internet research community.

**Figure 1:**
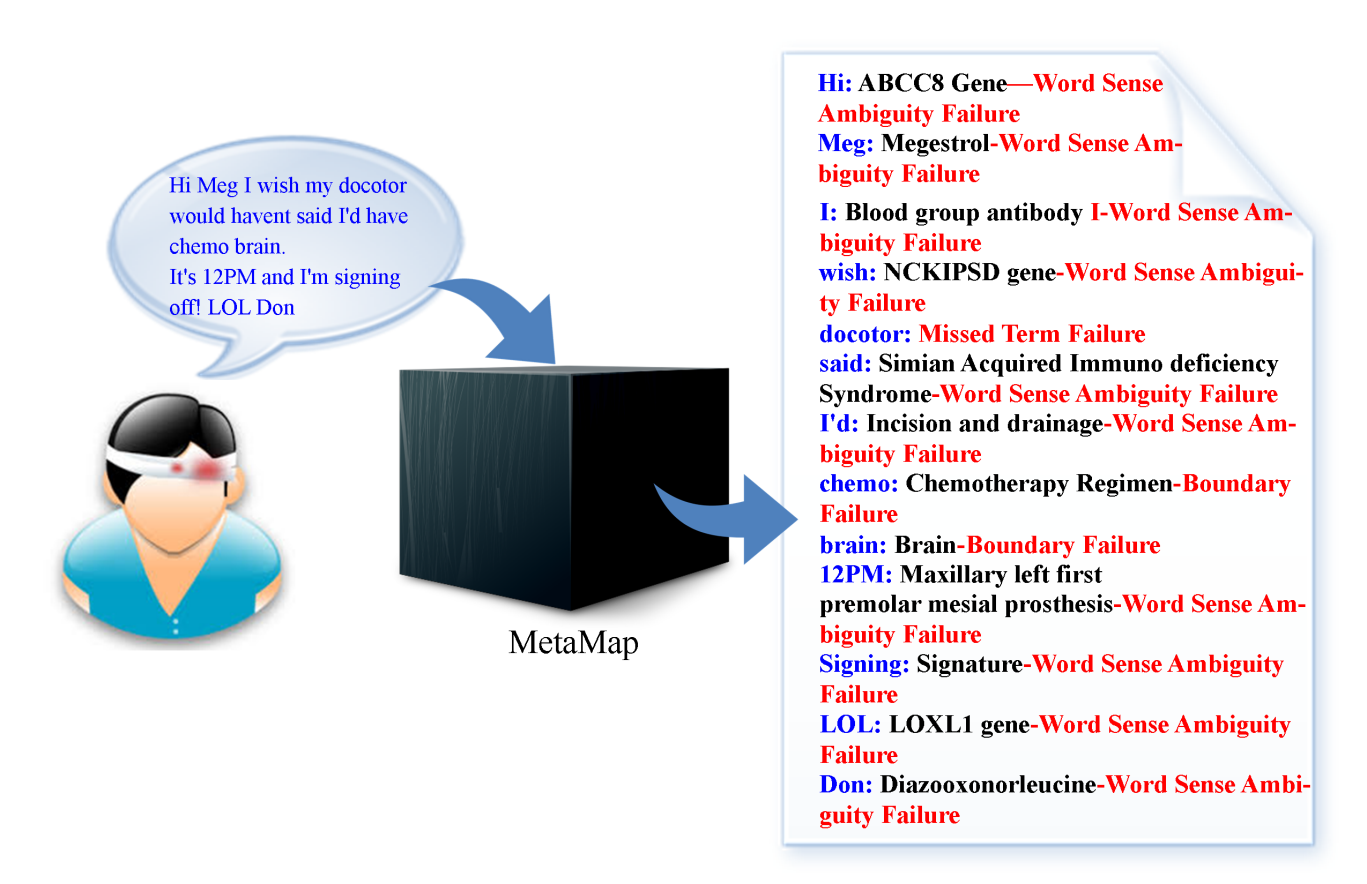
Example failures that resulted from the application of MetaMap to process patient-generated text in an online health community (blue terms represent patient-generated text; black terms represent MetaMap’s interpretation; and red terms represent failure type).

Figure 1 illustrates the challenges of processing patient-generated online health community text and the common failures of biomedical NLP tools on that text. In an example sentence, “Hi Meg, I wish my docotor would haven’t said I’d have chemo brain. It’s 12PM and I’m signing off! LOL Don”, MetaMap produced 12 mappings, all of which were incorrect, and overlooked one misspelled term, “docotor”, thus producing 13 failures.

Some of these failures are already known problematic failures of MetaMap [26,29,34-36]. Our findings extend prior work by identifying the causes for each failure type. Leveraging our understanding of those causes, we developed automated techniques that identified these previously highlighted failures effectively without having to produce manually annotated datasets. In demonstrating the feasibility of our automated failure detection tool, we delineated the use of easily accessible NLP techniques and dictionaries. These techniques can independently examine each failure type. We provided a detailed demonstration of our failure detection tool to allow researchers to select the parts of our approach that meet the focus of their NLP tool assessment. Additionally, our detection approach can be modified and used to rectify failures in NLP tools.

We focused our research on MetaMap; however, findings from our study can apply to other NLP tools in a similar manner. Few failure causes, such as inconsistency of word sense disambiguation feature, pertain more to MetaMap than other tools. However, any NLP tools that provide semantic connections require a similar word sense ambiguity feature. Moreover, different NLP tools could excel in different areas, and our automated failure detection can cost-effectively highlight problematic areas. Similarly, our techniques for detecting failures could strengthen the performance of other NLP tools to process patient-generated text and more traditional types of text. For instance, the word sense ambiguity failure caused by neglecting POS information can also be problematic in different types of text, including biomedical literature. That failure might surface less frequently due to differences in sentence structure between the biomedical literature and patient-generated text. Nevertheless, it is a significant problem that applies to both types of text. Applying such POS information when mapping a term could increase the accuracy of the mappings from a variety of texts. Another example is the missed term failure caused by community nomenclature. MetaMap or other NLP tools will miss terms if particular synonyms are missing from the vocabulary source. Researchers could use the algorithm by Schwartz and Hearst [46] to collect various synonyms that are used in different domains and frequently update the vocabulary sources, such as UMLS. Furthermore, researchers could use the splitting-a-phrase detection technique to not only prevent boundary failures, but collect new medical jargon (eg, “chemo curls”) and identify important concepts missing in the UMLS (eg, “double mastectomy”).

The dictionary-based matching and NLP techniques used in our detection process were evaluated in previous studies [44-46]. However, these studies were conducted in different domains and have been shown to produce errors. Moreover, these tools were not evaluated for patient-generated text. In addition, the automated detection techniques are generally limited to the coverage of the UMLS and MetaMap’s capability to map when accurate and full spellings were provided. To strengthen our findings, we evaluated each detection method as well as the overall performance (Table 4). However, our findings could be biased towards cancer community text and could be further strengthened by generalizing our results in different platforms or patient groups. It is also plausible that we have not encountered all failure types or causes for other patient-generated health data contexts.

Moreover, a number of updates were made for both the UMLS and MetaMap [51] since the beginning of our study. To maintain consistency, we continued to use the same versions of the UMLS and MetaMap. However, we used the latest version of MetaMap (2013) and the UMLS (2013AB) to process a sample of 39 posts that were illustrated here and then compared the results to our findings. Although some of these causes were amended in the new version of MetaMap, the majority (33/39, 85%) of the outcomes remained unchanged or changed but still problematic. All the improvements (6/39, 15%) were word sense ambiguity failures. The improved cases included (1) colloquial languages, (2) email addresses and URLs, (3) Internet slang and SMS language, (4) mismapped verbs, and (5) two cases of inconsistent mappings. Despite these improvements, none of the described 12 causes of failures had been completely addressed. The lack of significant improvement further illustrated the magnitude of the challenges of processing patient-generated text. Because technologies, source vocabularies, and characteristics of text continue to be updated in the field of NLP, the need for low-cost automated methods to assess the updates will continuously increase. We demonstrate the feasibility of such automated approaches in detecting common failures using MetaMap and patient-generated text.

Although our study focused on online health community text, the insights inform efforts to apply NLP tools to process various types of patient-generated text, including blogs or online journals, which share similar narrative writing styles and colloquial language. Moreover, Facebook and email provide conversational interactions similar to the interaction in online health communities. Tweets about emergency responses [5], public health trends [52], or clinical notes from electronic medical records (EMR) could contain a host of abbreviations that NLP tools could incorrectly map. Thus, our failure detection techniques could be applied in these other contexts to assess the capability of processing different types of patient-generated text.

### Conclusion

Processing patient-generated text provides unique opportunities. However, this process is fraught with challenges. We identified three types of failures that biomedical NLP tools could produce when processing patient-generated text from an online health community. We further identified causes for each failure type, which became the basis for applying automated failure detection methods using pre-validated NLP and dictionary-based techniques. Using these techniques, we showed the feasibility of identifying common failures in processing patient-generated health text, at a low cost. The value of our approach lies in helping researchers and developers quickly assess the capability of NLP tools for processing patient-generated text.

## Abbreviations

CHV: Consumer Health Vocabulary
cTakes: Clinical Text Analysis and Knowledge Extraction System
CUI: concept unique identifiers
EC: esophageal cancer
EMR: electronic medical records
LOL: laugh out loud or lots of love
MedLEE: Medical Language Extraction and Encoding System
NLM: National Library of Medicine
NLP: natural language processing
POS: part of speech
Rad: radiation absorbed dose
SAID: Simian Acquired Immunodeficiency Syndrome
SKR: semantic knowledge representation
SMS: short message service
UMLS: Unified Medical Language System
URL: Uniform Resource Locator
XOXO: hugs and kisses

## References

[1] Fox S, Rainie L (2014). Pew Research Center Internet, Science & Tech.

[2] Fox S (2005). Pew Research Center Internet, Science & Tech.

[3] Fox S (2011). Pew Research Center Internet, Science & Tech.

[4] Eysenbach G (2008). Medicine 2.0: social networking, collaboration, participation, apomediation, and openness. J Med Internet Res.

[5] Starbird K, Palen L (2011). ‘Voluntweeters’: Self-Organizing by Digital Volunteers in Times of Crisis. http://dl.acm.org/citation.cfm?id=1979102.

[6] Wicks P, Vaughan TE, Massagli MP, Heywood J (2011). Accelerated clinical discovery using self-reported patient data collected online and a patient-matching algorithm. Nat Biotechnol.

[7] Wicks P, Massagli M, Frost J, Brownstein C, Okun S, Vaughan T, Bradley R, Heywood J (2010). Sharing health data for better outcomes on PatientsLikeMe. J Med Internet Res.

[8] Frost JH, Massagli MP (2008). Social uses of personal health information within PatientsLikeMe, an online patient community: what can happen when patients have access to one another's data. J Med Internet Res.

[9] Wen M, Rose C (2012). Understanding participant behavior trajectories in online health support groups using automatic extraction methods. http://dl.acm.org/citation.cfm?id=2389205.

[10] Wang Y, Kraut R, Levine JM (2012). To Stay or Leave ? The Relationship of Emotional and Informational Support to Commitment in Online Health Support Groups.

[11] Chee BW, Berlin R, Schatz B (2011). Predicting adverse drug events from personal health messages. AMIA Annu Symp Proc.

[12] MacLean DL, Heer J (2013). Identifying medical terms in patient-authored text: a crowdsourcing-based approach. J Am Med Inform Assoc.

[13] Maclean DL, Gupta S, Lembke A, Manning C, Heer J (2015). Forum77: An Analysis of an Online Health Forum Dedicated to Addiction Recovery.

[14] De Choudhury M, Gamon M, Counts S, Horvitz E (2013). Predicting depression via social media. http://www.aaai.org/ocs/index.php/ICWSM/ICWSM13/paper/viewFile/6124/6351.

[15] Huh J, Yetisgen-Yildiz M, Pratt W (2013). Text classification for assisting moderators in online health communities. J Biomed Inform.

[16] Savova GK, Masanz JJ, Ogren PV, Zheng J, Sohn S, Kipper-Schuler KC, Chute CG (2010). Mayo clinical Text Analysis and Knowledge Extraction System (cTAKES): architecture, component evaluation and applications. J Am Med Inform Assoc.

[17] Musen MA, Noy NF, Shah NH, Whetzel PL, Chute CG, Story M, Smith B (2012). The National Center for Biomedical Ontology. J Am Med Inform Assoc.

[18] Jonquet C, Shah NH, Musen MA (2009). The open biomedical annotator. Summit on Translat Bioinforma.

[19] Friedman C, Hripcsak G, DuMouchel W, Johnson SB, Clayton PD (2008). Natural language processing in an operational clinical information system. Nat Lang Eng.

[20] Friedman C (1997). Towards a comprehensive medical language processing system: methods and issues. Proc AMIA Annu Fall Symp.

[21] Chen L, Friedman C (2004). Extracting phenotypic information from the literature via natural language processing. Stud Health Technol Inform.

[22] Aronson AR, Lang F (2010). An overview of MetaMap: historical perspective and recent advances. J Am Med Inform Assoc.

[23] Humphreys BL, Lindberg DA, Schoolman HM, Barnett GO (1998). The Unified Medical Language System: an informatics research collaboration. J Am Med Inform Assoc.

[24] Chen Y, Perl Y, Geller J, Cimino JJ (2007). Analysis of a study of the users, uses, and future agenda of the UMLS. J Am Med Inform Assoc.

[25] Zeng Q, Kogan S, Ash N, Greenes RA (2001). Patient and clinician vocabulary: how different are they?. Stud Health Technol Inform.

[26] Smith CA, Wicks PJ (2008). PatientsLikeMe: Consumer health vocabulary as a folksonomy. AMIA Annu Symp Proc.

[27] Keselman A, Smith CA, Divita G, Kim H, Browne AC, Leroy G, Zeng-Treitler Q (2008). Consumer health concepts that do not map to the UMLS: where do they fit?. J Am Med Inform Assoc.

[28] Zeng QT, Crowell J, Plovnick RM, Kim E, Ngo L, Dibble E (2006). Assisting consumer health information retrieval with query recommendations. J Am Med Inform Assoc.

[29] Brennan PF, Aronson AR (2003). Towards linking patients and clinical information: detecting UMLS concepts in e-mail. J Biomed Inform.

[30] Smith CA, Stavri PZ, Chapman WW (2002). In their own words? A terminological analysis of e-mail to a cancer information service. Proc AMIA Symp.

[31] Zeng QT, Tse T (2006). Exploring and developing consumer health vocabularies. J Am Med Inform Assoc.

[32] Elhadad N, Zhang S, Driscoll P, Brody S (2014). Characterizing the Sublanguage of Online Breast Cancer Forums for Medications, Symptoms, and Emotions.

[33] Strauss Al, Corbin J (1990). Basics of qualitative research. Basics of qualitative research: grounded theory procedures and techniques.

[34] Divita G, Tse T, Roth L (2004). Failure analysis of MetaMap Transfer (MMTx). Stud Health Technol Inform.

[35] Pratt W, Yetisgen-Yildiz M (2003). A study of biomedical concept identification: MetaMap vs. people. AMIA Annu Symp Proc.

[36] Kang N, Singh B, Afzal Z, van Mulligen EM, Kors JA (2013). Using rule-based natural language processing to improve disease normalization in biomedical text. J Am Med Inform Assoc.

[37] Danescu-Niculescu-Mizil C, West R, Jurafsky D, Leskovec J, Potts C (2013). No Country for Old Members: User Lifecycle and Linguistic Change in Online Communities.

[38] McCray AT, Ide NC, Loane RR, Tse T (2004). Strategies for supporting consumer health information seeking. Stud Health Technol Inform.

[39] McCray AT, Ide NC (2000). Design and implementation of a national clinical trials registry. J Am Med Inform Assoc.

[40] Smith L, Rindflesch T, Wilbur WJ (2004). MedPost: a part-of-speech tagger for bioMedical text. Bioinformatics.

[41] Jansen E, James V (2002). NetLingo: the Internet dictionary.

[42] Bureau U census (1990). Frequently Occurring Names in the US.

[43] Bamford S, Dawson E, Forbes S, Clements J, Pettett R, Dogan A, Flanagan A, Teague J, Futreal PA, Stratton MR, Wooster R (2004). The COSMIC (Catalogue of Somatic Mutations in Cancer) database and website. Br J Cancer.

[44] Toutanova K, Klein D, Manning C, Singer Y (2003). Feature-Rich Part-of-Speech Tagging with a Cyclic Dependency Network. http://dl.acm.org/citation.cfm?id=1073478.

[45] De Marneffe M-C, MacCartney B, Manning CD (2006). Generating Typed Dependency Parses from Phrase Structure Parses.

[46] Schwartz AS, Hearst MA (2003). A simple algorithm for identifying abbreviation definitions in biomedical text. Pac Symp Biocomput.

[47] Chapman WW, Bridewell W, Hanbury P, Cooper GF, Buchanan BG (2001). A simple algorithm for identifying negated findings and diseases in discharge summaries. J Biomed Inform.

[48] Baumgartner WA, Lu Z, Johnson HL, Caporaso JG, Paquette J, Lindemann A, White EK, Medvedeva O, Cohen KB, Hunter L (2008). Concept recognition for extracting protein interaction relations from biomedical text. Genome Biol.

[49] Jimeno-Yepes A, Berlanga-Llavori R, Rebholz-Schuhmann D (2010). Ontology refinement for improved information retrieval. Information Processing & Management.

[50] Query Suggestion Service.

[51] MetaMap Updates.

[52] Dredze M (2012). How Social Media Will Change Public Health. IEEE Intell. Syst.

